# Applying a new theoretical and methodological approach for behavior-change campaign planning: identifying the critical determinants for reducing littering and evaluating the resulting large-scale campaign

**DOI:** 10.3389/fpsyg.2024.1441094

**Published:** 2024-10-15

**Authors:** Robert Tobias, Nicole Moraz, Barbara Degenhardt

**Affiliations:** ^1^Social Psychology, Department of Psychology, University of Zurich, Zurich, Switzerland; ^2^Irchel Campus Usage Management, University of Zurich, Zurich, Switzerland

**Keywords:** littering, pro-environmental behavior, behavior change, intervention, quasi-experiment/field study, parks/trails, behavior determinants, information campaign

## Abstract

**Introduction:**

This paper presents a theoretical concept and methodological approach for identifying critical determinants for behavior change interventions. The approach is based on established theories and constructs but represents them in an intervention- instead of questionnaire-oriented form. Six discriminant and targetable dimensions of behavior determinants are proposed: Consideration, feasibility, instrumental evaluation, norms and goals, affective evaluation, and needs and tension states.

**Methods:**

For estimating the importance of these dimensions for a specific behavior to be changed in a specific situation and population, a quasi-experimental approach is proposed, in which interventions are designed to have effects on one of these dimensions but none on the other dimensions. By measuring changes of the target behavior or its consequences, the impact of each dimension on changing the behavior can be estimated *in-situ* without questionnaires. The approach was applied to develop a campaign for reducing picnic littering in an urban park in Zurich (Switzerland). In 2019, posters targeting four dimensions were set up during three waves in up to four zones with two control zones without posters. Before, between, and after the intervention waves, for at least 2 weeks, no interventions were in place. The volume of litter was measured on 119 days at 55 points.

**Results:**

In some cases, the amount of litter was too small for effects to be detected, but where enough littering occurred, posters providing information, inducing positive emotions, or activating reciprocity norms—as well as providing the option of separating fractions of waste for recycling as a structural measure—reduced litter significantly. Interventions targeting the tension state of disgust had no effect. Posters targeting descriptive and injunctive norms increased the amount of litter.

**Discussion:**

Based on the results of the preparative study, a large-scale campaign was designed, implemented, and evaluated in 2022, which led to promising effects.

## 1 Introduction

Based on decades of psychological research, it is well documented what hinders people from changing problematic behaviors, and what behavior-change techniques could be applied in principle. However, it turned out to be difficult to apply this accumulated knowledge on behavioral determinants, mediators and moderators to specific real-world settings or interventions. That is, general recommendations about when to apply which technique in what form has shown not being enough to deal with the complexity and dynamics of real-world problems. Tools are required that—based on scientific evidence and empirical data, and considering the challenges of applied settings—help determine barriers of and design behavior-change techniques for specific situations, populations, and behaviors (c.f. Michie et al., 2011; Weaver, 2015). In this paper, we will present, apply, and discuss a new approach that serves this function, using the example of littering.

The impact of litter (i.e., misplaced solid waste, Geller, 1980) is dramatic on many levels. As an environmental problem, it directly harms wildlife and humans alike. For example, it can cause injuries, poisoning, attract vermin, or decompose to dangerous substances (Berger et al., 2008). But as with most environmental problems, littering can also have economic impacts. For example, it creates aesthetic issues (Pandey, 1990) that reduce the recreational and property value of affected areas (Skogan, 1990). Even social problems can be exacerbated by littering. Litter has a symbolic effect indicating the absence of care and the acceptance of transgressions, which can lead to an increase in social transgressions like theft (Keizer et al., 2008).

Litter causes so many problems that the reduction of littering was one of the first topics of environmental protection psychology, which started in the sixties, and much research was done to inform litter prevention programs (Burgess et al., 1971; Cone and Hayes, 1980; Geller et al., 1982). In a systematic review, Chaudhary et al. (2021) identified 70 articles on littering behavior published between 1971 and 2018. A summary of this research is also provided by Schultz et al. (2013). They identified three approaches to littering research: (1) the search for the demographic characteristics of people who litter, (2) the effect of the physical context on littering behavior, and (3) the analysis of what is littered.

Early investigations into psychological processes behind littering mostly focused on normative signals of the situations in which littering happens, such as litter that is already present (Cialdini et al., 1990) or how ‘disordered’ the setting is (Keizer et al., 2008). The conclusion drawn from these studies was that litter can best be prevented by conveniently placing garbage bins and removing any litter as quickly as possible (Schultz et al., 2013; Van Doesum et al., 2021). This ‘solution’ is, however, often not satisfying. More frequent cleaning and providing more garbage bins—which need to be emptied—can be more costly than collecting more litter. Furthermore, a larger number of conveniently placed garbage bins may reduce the recreational value of a landscape (e.g., Van Doesum et al., 2021).

According to Schultz et al. (2013), only 15% of all littering acts result from contextual variables. Therefore, littering-reducing measures that target psychological processes might be a more efficient approach to solve the littering problem. Such interventions require a deeper understanding of these psychological processes. One approach to investigating psychological processes is with questionnaires (e.g., Ai and Rosenthal, 2024; Mori et al., 2024; Oduro-Appiah et al., 2024; Ojedokun et al., 2022). The most influential constructs in questionnaire-based studies are related to normative evaluations and the awareness of consequences or ascription of responsibility. However, while survey-based investigations allow a precise assessment of psychological constructs and can be informative for the investigation of many behaviors, in the case of littering, such data has a limited value. Not only is littering a socially undesired or even illegal behavior, for which strongly biased answers must be expected. More importantly, most forms of littering are not a result of deliberate decisions. Still, people can be aware of the behavior and conscious about their decision in the situation. But people might not reflect on their reasons and therefore not be able to explain what happened in the situation. We must assume that retrospectively assessed reports are constructed in the moment of answering the questionnaire items and not necessarily reflect the processes determining the behavior in the situation. This is well-illustrated by Hansmann and Steimer’s (2017) study where participants provided very different reasons for their own littering and the littering of other people. Also, in the study of Farage et al. (2021), the littering-avoidance intention, which was assessed with questionnaire items, could be explained well, while the observed littering could not be explained.

For a behavior that cannot be investigated well with questionnaire data, experimental methods are indicated. However, experiments in the laboratory are also problematic. Littering usually has a strong situational component, making it difficult to extrapolate from laboratory or online settings to the real world. Therefore, we conclude that field experiments with interventions on various psychological processes and measurement of the actual littering (e.g., Gangl et al., 2022) lead to the most valid information on how littering can be reduced. This form of investigation is, however, sparce (Chaudhary et al., 2021). Furthermore, often artificial forms of littering, such as distributed flyers (e.g., Hansmann and Steimer, 2016), are used that might not well represent natural occurring littering.

The present study starts from this idea of investigating the psychological processes driving littering or rather littering reduction with field experiments. The goal is to identify the psychological constructs or processes that are most promising to be targeted in a large-scale anti-littering campaign—to generate an empirical basis for designing anti-littering measures for a specific location and population and develop the intervention material with this empirical basis. For such an endeavor, several research gaps need to be tackled.

First, littering behavior needs to be better specified from a psychological perspective. While from an environmental perspective, the critical aspects are what is littered where, from a psychological perspective, litter is a consequence of a variety of behaviors that can have different psychological characteristics. This paper proposes a rough classification of some forms of littering and specifies the type of littering investigated.

Second, psychological constructs are mainly specified by questionnaire items and for many, it is difficult to design manipulations that target all facets of the construct without affecting other constructs. Therefore, the present paper will propose a theoretical concept that represents established psychological constructs from an intervention instead of a questionnaire perspective.

Third, most studies that investigate littering behavior stop with quantifying effects. However, it is still a huge step from such results to a large-scale campaign. This paper exemplifies, for a specific case, how results of a scientific study can be used to develop a campaign—and estimates the effect of such an intervention, considering the limited resources available in non-scientific projects. Finally, this paper adds to the growing body of evidence on psychological determinants of littering and litter-avoidance behavior and presents the effects of a large-scale litter-reduction campaign.

## 2 Theoretical background

### 2.1 Behavior specification and study approach

The many behaviors that can lead to misplaced solid waste can be driven by very different psychological processes. Therefore, for designing psychological measures, it is necessary to specify what behaviors shall be changed. Personal site inspections and interviews with the Operational and Security Services revealed that four types of littering were prevalent in the investigated park: (1) Careless disposal of small pieces of garbage, particularly cigarette butts. This form of littering is strongly determined by habits, and campaigns targeting such behavior need to focus on measures to prepare for or change the situations where such behavior could potentially happen (e.g., providing more ashtrays, which also serve as memory aids). (2) Leaving behind garbage after a prolonged stay at a place, e.g., after a picnic. This form of littering can occur for a variety of reasons, but measures targeting such behavior should have a direct effect, because the interventions used can be perceived and processed before littering is irreversible. (3) Particularly at night, parties are common in the park that often result in larger amounts of litter. While the causes for this form of littering can be like for picnic littering, further factors might play a role. In some cases, littering might be intentional to demonstrate rejection of norms or show the world how wild the party was. In other cases, people might have been so intoxicated that they were unable to clean up even if they had intended to do so. (4) The park is sometimes also used to dispose of larger amounts of garbage from households or even construction sites. This is finable and, due to its different legal status, such behavior is often not considered littering but illegal garbage disposal.

For this study, the second form of littering (‘picnic littering’) was selected because measures to change such behavior are easier to implement and picnic littering is easier to measure: It can be (mostly) isolated from careless litter disposal by limiting the measurement of litter to places where people stay longer. Picnic littering can also be separated from party littering by limiting the measurement to the afternoon because the park is cleaned early in the morning and parties happen mostly at night.

Once the to-be-changed behavior is specified, the main task is to identify the psychological processes that should be targeted to induce the desired changes most efficiently. In many cases, a survey that assesses psychological constructs, the behavior, and further information could be used. However, for campaign planning, surveys have several disadvantages. Particularly, it is difficult to reach even a roughly representative sample of the target population and the relation between questionnaire data and intervention effects is only based on theoretical assumptions—not to speak of possible biases in the questionnaire answers. In the case of poorly reflected behavior decisions, such as we assume for littering, little useful information can be deduced from questionnaire data. Neither do people elaborate much about the decisions to leave waste behind, nor do they remember what drove their behavior, when asked about it later. We expect the answers to be mostly defensive post-hoc rationalizations, such as a supposed lack of garbage bins.

Therefore, within the development of a real-world campaign to reduce behaviors such as littering, a quasi-experimental field study appears to be the best approach. It allows identifying critical psychological processes without questionnaires, in the specific situation and representatively from the target population. To avoid ethical issues, ideally, such an approach also omits direct observations of the behavior. Of course, field experiments come with their own set of challenges, particularly the limited controllability of the situation and participants. Effects could be moderated by unknown situational influences and experimental manipulations could have effects in other zones than they were installed. Such factors need to be considered in the interpretation of the data.

To conclude, the basic idea of the approach presented here is to conduct a quasi-experimental field study with (a) a systematic variety of interventions and (b) measure behavioral effects, i.e., the amount of litter, as outcome variable. Each intervention is designed to target only one psychological process or class of similar processes that are assumed to be determinants of the investigated behavior. From the comparison of the effects of these interventions, the relevance of each process for reducing littering can be derived.

### 2.2 Psychological determinants of behaviors

Psychology offers many theories and models to explain individual behaviors, such as the Theory of Planned Behavior (TPB; Ajzen, 1991), the Health Action Process Approach (HAPA; Schwarzer, 2008), or the Norm Activation Model (NAM; Schwartz, 1977), to name a few (*cf.* Hagger et al., 2020; Rau et al., 2022). Many researchers extended or combined the mentioned models (e.g., Bamberg, 2013; Klöckner and Blöbaum, 2010). However, on the abstract level as the concepts are discussed here, these models use the same psychological constructs. Therefore, in the following, we mainly refer to the constructs of the first three models. All these models proved valuable in multiple questionnaire-based studies and applications, however, many of their constructs cannot be used in an intervention-oriented approach as presented here.

First, many constructs are defined in overly general terms comprising several processes at once. For example, attitudes of the TPB comprise cognitive and affective processes, and outcome expectations of the HAPA even normative aspects, which are all different regarding their effects on the behavior and the form of interventions targeting them. For our study, it is necessary to separate processes that require different forms of interventions (*cf.* Hagger et al., 2020; Rau et al., 2022).

Second, between the models, differences of the constructs are often too subtle for interventions to be distinguished (*cf.* Hagger et al., 2020). For example, it would be almost impossible to design an intervention that changes perceived behavior control (a TPB construct) but not self-efficacy (a HAPA construct). Therefore, if constructs are specified by the type of interventions instead of wording of questionnaire items, such similar constructs are assumed to represent the same psychological process.

Third, each behavior model was designed with a specific perspective and thus, abstracts from certain processes that are not in the models’ focus. Particularly, processes that are difficult to assess by questionnaire (e.g., tension states) or that were assumed to be irrelevant due to the design of the studies testing the models, (e.g., consideration of the behavior), are often omitted. To have an optimal basis to select interventions, we will consider such psychological processes—at least as processes that should not be affected by interventions on other processes.

**Figure 1:**
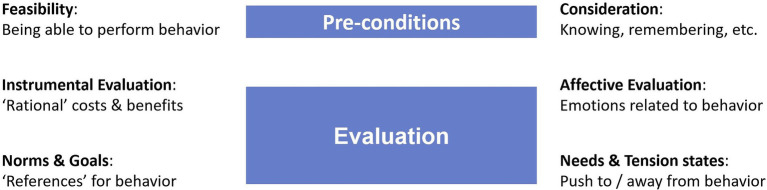
Dimensions of behavior determinants.

To guide the design of such specific interventions, we propose a set of dimensions of (social-) psychological behavior-determining processes (see Figure 1) derived from established behavior models, partially by abstraction and partially by specification. The idea is to group together psychological processes that are similar regarding the form of interventions targeting them, and separate processes that are different and often conflict with the processes of other dimensions. So, these dimensions are not single constructs, but classes of psychological processes. For a specific investigation, constructs and interventions targeting them need to be specified (e.g., monetary costs or injunctive norms). These constructs never cover an entire dimension of processes. However, not only can many constructs/interventions be specified within a dimension, but they can also cover several dimensions—such as attitudes or most interventions in real-world behavior-change campaigns. Such multi-dimensional interventions would, however, not be suitable for the study approach explained above.

This space of psychological determinants of behaviors is not meant to replace any behavior model. The main goal is to support the reflection of possible barriers of behavior change within a complex applied problem. Even if working with established constructs and measurement tools, for selecting them, one first needs to realize in more general terms, what could influence a behavior. The selection of investigated constructs and, thus, measurement tools should be based on a solid analysis of the problem and not, as is often done, the problem squeezed into a form that allows investigating the favored constructs. Within the approach presented in this paper, such general space of determinants is particularly essential, because not only *the processes targeted* by the interventions need to be specified, but also what processes *must not be affected* by these interventions. While it is impossible to specify all constructs that shall not be affected by an intervention, a rough system of dimensions allows designing focused interventions to inform the development of real-world campaigns.

We propose six dimensions of processes involved in behavior selection, which open two distinct spaces, one of two dimensions—the pre-conditions of behavior execution—and one of four dimensions—the evaluation of behavior execution (Figure 1). The pre-conditions consist of *consideration* and feasibility. For a behavior option to be evaluated, it must be considered (i.e., known, remembered, and seen as adequate in a specific situation). Consideration does not require consciousness, neither of the behavior options, nor of the process of selecting an option. This becomes particularly apparent in the case of habits which, according to Tobias (2009), act also on consideration: If we have a strong habit for a behavior in a specific situation, we might enact it without even remembering having done such action. However, the habit led to considering the selected option. Besides investigations into prospective memory (e.g., McDaniel and Einstein, 2007), in most models explaining behavior, such as the TPB or the HAPA, consideration is either not separated from feasibility or not considered at all. Most studies assume consideration of the behavior under investigation as a given. However, knowledge about how to perform the behavior or about the behavior’s consequences are often investigated and targeted in studies and campaigns (e.g., Hossain et al., 2022; Liu et al., 2020).

*Feasibility* is found in most behavior models in one or another form, representing a perceived external situational factor that makes a behavior more or less difficult or controllable to perform (c.f. perceived behavior control in the TPB; self-efficacy in the HAPA). However, these constructs are conceptualized differently than in our approach, mainly representing any situational effect that might lead to a discrepancy of a previous evaluation (resulting in an intention) and the actual behavior performance. In contrast, in our approach, any dimension of behavior determinants can be personal or situational (or related to the population^1^). Feasibility is conceptualized as the perception that a behavior is possible to perform. This is distinguished from the effort to perform a behavior, which is part of the evaluation explained later. For example, if people form their intentions of the travel mode to use for commuting, they might deem public transportation not feasible, because there exists no public transportation at their location. Here, feasibility acts as a personal barrier to using public transportation. However, if they have public transportation options, but deem them too expensive or time consuming to use, it would not be feasibility, but rather instrumental evaluation (see below) that acts as a personal barrier. Equally, feasibility as well as instrumental evaluation can act as situational barriers. If a person usually travels by public transport but, on one day, it is cancelled, using this travel mode is considered not feasible in this situation. Or, this person wants to get home faster, one day, and, thus, instrumental evaluation acts as a situational barrier to using public transportation. In most established behavior models, in the situation, no distinction between feasibility and evaluations is made and all situational influences subsumed in constructs, such as low perceived behavior control or self-efficacy. In contrast, here, the same distinction between feasibility and evaluation is made for barriers when forming the intention or when performing the behavior.

Behavior options that a person considers and deems feasible are evaluated in a space of four dimensions: (1) instrumental evaluation, (2) norms and goals, (3) affective evaluations, and (4) needs and tensions states. *Instrumental evaluation* expresses the extent to which performing a behavior is worthwhile, considering costs and benefits. This dimension comprises resources such as money and time, but also consequences of the behavior, for example, for health or the environment. Such cost–benefit evaluations are found in most psychological models for explaining behaviors, but mostly they are combined with other dimensions proposed here. For example, the attitudes of the TPB comprise instrumental and affective aspects (Breckler and Wiggins, 1989). The outcome expectations of the HAPA comprise all consequences of performing a behavior, not only instrumental, but also affective and normative (Schwarzer, 2008). Instrumental evaluation depends strongly on an individual’s mental models related to the behaviors. For example, how behavior performance impacts the environment or who is responsible for doing something about it. Thus, instrumental evaluation comprises processes related to comparisons of advantages and disadvantages of behavior options and their consequences.

(2) *Norms and goals* comprise the references to which individuals compare behaviors and behavior consequences. Many theories consider norms referring directly to the behaviors, such as descriptive and injunctive norms (Cialdini et al., 1990). They express how many others are observed or perceived performing the behaviors (descriptive norms) and how good or bad other people perceive the behaviors (injunctive norms). For the NAM, norms are the main drivers of behaviors. In the TPB, such norms are considered as construct in their own right (subjective social norms, Ajzen, 1991), while, in the HAPA, they are a part of the outcome evaluations (Schwarzer, 2008). Another norm considered in our study is reciprocity: People feel obligated to return a favor (Johnson et al., 1989). However, norms are not the only constructs used as reference to evaluate behaviors and their consequences. The goals a person pursues are also important (Brandstätter and Bernecker, 2022). Norms and goals also comprise symbolic functions of behaviors—in the sense of Dittmar (1992) for material possessions—which means that some behaviors are not only done for their instrumental consequences, but also for the meaning they express (e.g., driving a large car to appear as rich and powerful, Wicklund and Gollwitzer, 1981). To conclude, norms and goals comprise processes of evaluating the fit of behavior options and their consequences to references in the social environment or own standards.

Instrumental evaluation and norms and goals require a certain level of deliberation. However, some evaluations can be done almost spontaneously, such as (3) the *affective evaluation*. This dimension expresses how much a person feels like doing the behavior or perceives the behavior execution as pleasant or annoying. Most theories do not consider affective evaluation as an individual factor and combine it with instrumental evaluation (e.g., attitudes in the TPB or outcome evaluations in the HAPA). However, instrumental evaluation and affective evaluation are based on different psychological processes, show different dynamics (e.g., affective evaluation fluctuates faster and depends more on situational influences), and often lead to antagonistic evaluations (e.g., taking garbage to the garbage bin might be annoying with reference to affective evaluation, but better for the environment with reference to instrumental evaluation). Affective evaluation cannot be changed with arguments but mainly builds on emotional experiences and their associations with behaviors, forming affective connotations of these behaviors. However, behaviors can also have intrinsic characteristics that make them more or less enjoyable to perform. Based on this, behaviors can also serve affective functions and are performed, for example, to improve one’s mood or relax and reduce stress (Degenhardt and Buchecker, 2012; Gatersleben and Steg, 2013). Therefore, affective evaluation comprises processes of experiencing a desire or aversion to performing a behavior. For the behavior selection, particularly the feelings experienced when thinking about the behavior options before and while selecting them are critical. Note that expected emotions are rather part of the instrumental evaluation (or between the instrumental and affective evaluation), because expected emotions are not experienced but considered as an advantage or disadvantage of performing a behavior.

The last evaluation dimension, (4) *needs and tension states*, is rarely considered as its own factor in models, studies, or campaigns, but rather combined with affective evaluation. However, regarding psychological processes and behavior-change techniques, there are important differences between these two dimensions. While affective evaluation determines behavior selection mainly when no other ‘good reasons’ for doing something exist, needs and tension states have a strong motivating aspect that can push people towards performing or avoiding certain behaviors. Needs and tension states comprise all ‘strong feelings’ (many of them can be assessed as specific emotions) that go beyond a mere valence on a scale of pleasant to unpleasant and are perceived as pushing towards or away from a behavior. These can be based on physical needs (e.g., hunger or strain), perceptions of the world (e.g., fear, guilt, disgust, dissonance, reactance, anger, envy, shame, or injustice), or be provoked intentionally (e.g., with an implementation intention; Gollwitzer and Sheeran, 2006) to align the situational behavior selection with strategic evaluations and goals. Most of these tension states form research topics of their own. For the present study, disgust is of particular interest (e.g., Aunger and Curtis, 2013; Curtis et al., 2004). Regarding behavior change techniques, affective evaluations can only be changed by emotional experiences related to the respective behavior. In contrast, tension states can also be changed by targeting cognitive processes with information and arguments.

All these dimensions are on a high level of abstraction and for each case, they need to be further specified. As already mentioned, within instrumental evaluation, costs of money and time or consequences for the environment or health could be important. Equally, within norms and goals, descriptive, injunctive or reciprocity norms could be distinguished. Which further specifications are used, distinguished, or combined depends on the requirements of each case. Often, however, despite the subtle differences, the psychological processes involved might be similar. Saving time might be done to save money, information about descriptive norms might be interpreted as an indicator for what people actually think is the right thing to do, etc. Of course, barriers are often formed from several dimensions and, for behavior-change campaigns, interventions mostly target various dimensions. However, for identifying the critical dimensions as basis for planning a campaign, interventions are required that target only one dimension.

Applying this intervention-oriented specification of behavior determinants to the approach presented in the previous sub-section (2.1), we can investigate the role of each determinant without using questionnaires. By applying manipulations that target only one dimension and affect other dimensions as little as possible, a field experiment can be used to estimate the role of each dimension for reducing littering. The effect of the interventions on the measured behavior or its consequences (e.g., amount of litter) indicates the importance of the respective dimension for changing the behavior. This method was used in preparation of a large-scale anti-littering campaign in an urban park in Zurich, Switzerland. The proposed intervention-oriented specification of established constructs should not be understood as a new theoretical model, but as a tool that makes the knowledge compiled in established behavior models better accessible to the design of campaigns. Therefore, this study does not present hypothesis tests but investigates how the proposed approach is useful for the design of campaigns. Of course, a single study is just a first step of such investigation and further applications of the proposed approach would be required to build confidence in the utility of the method.

## 3 Preparative investigation

### 3.1 Materials and methods

#### 3.1.1 Site and participants

**Figure 2:**
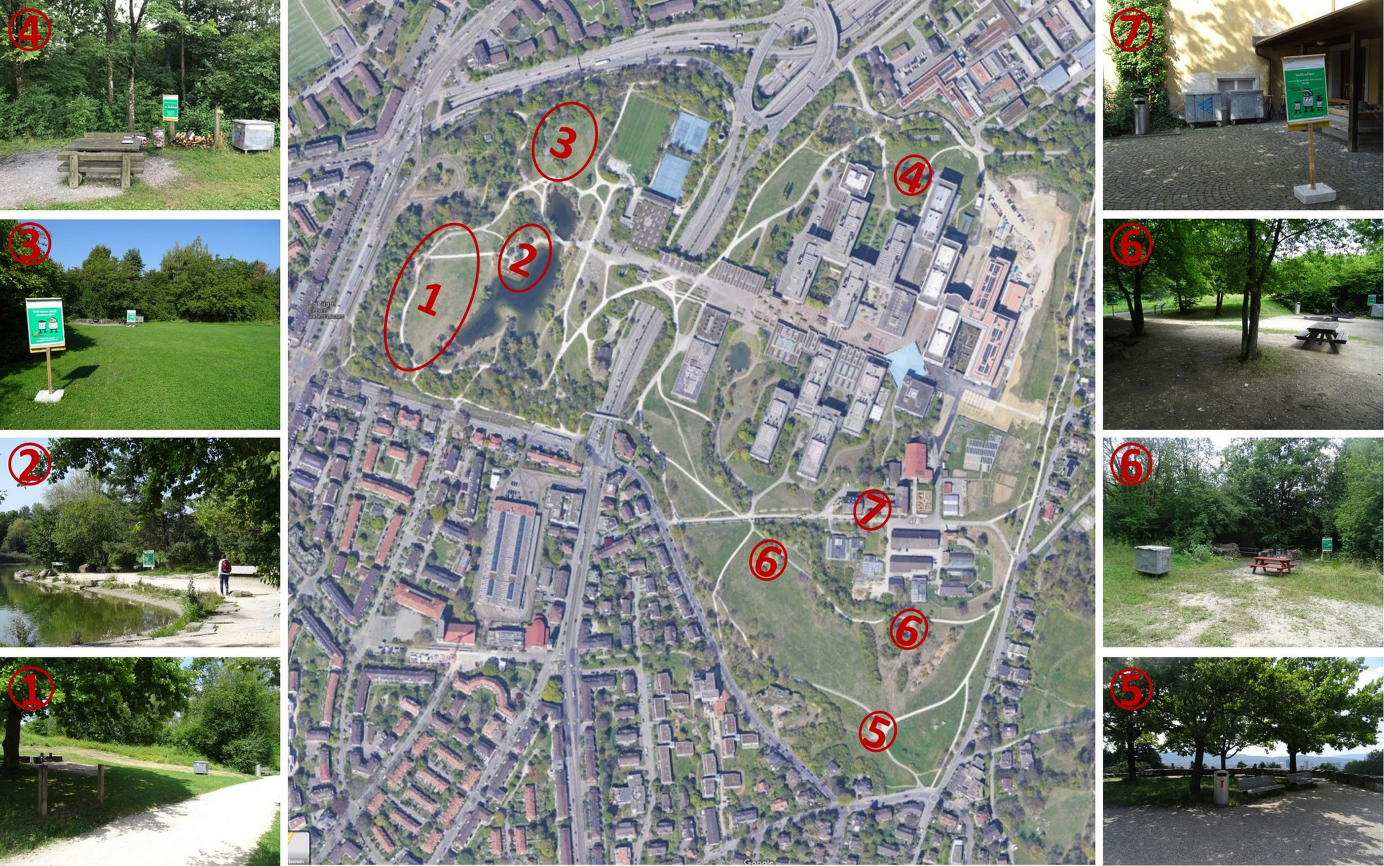
Study site (Irchelpark, source: Google Maps) with the investigation zones (photos by first author).

The investigation was done in the ‘Irchelpark’, an urban park in the north of the City of Zurich, Switzerland, with an extension of about 800 × 600 m (see Figure 2). This park was created in the 1980s as a nature-oriented recreational area and surrounds one of the campuses of the University of Zurich. While officially not a part of the university, maintenance of the park is done by personnel of the university. Due to its location, the park is highly frequented by a wide range of users, including students, inhabitants of the surrounding residential areas, people from the surrounding offices having a break, families making a picnic or barbecue, groups of adolescents throwing parties, and homeless people. The park has several ponds, offers options for sports, provides important transit routes for pedestrians, and frequently hosts large events. The park is also frequented by animals, including large birds and foxes, as one side continues into the woods, is relatively protected from the urban noise, and suffers from minimal light contamination.

In 2018, Irchel Campus Usage Management initiated a joint initiative with the university’s Operational Service unit that cleans the Irchelpark and the Chair of Social Psychology to develop measures for reducing littering in the Irchelpark. The size and strong segmentation of the park made the site well suited for the presented approach, as similar but clearly separated zones could be specified. In late 2018, project financing was secured, and the first part of the project—the investigation of the critical psychological dimensions—was designed and organized from the winter of 2018 to the beginning of 2019. Data gathering took place from May 6 to September 29, 2019, which comprised almost the entire warm period of that year. Unfortunately, the weather in May 2019 was unusually cold and wet, so, in the beginning of the study, the park was not visited often.

#### 3.1.2 Experimental manipulations

**Figure 3:**
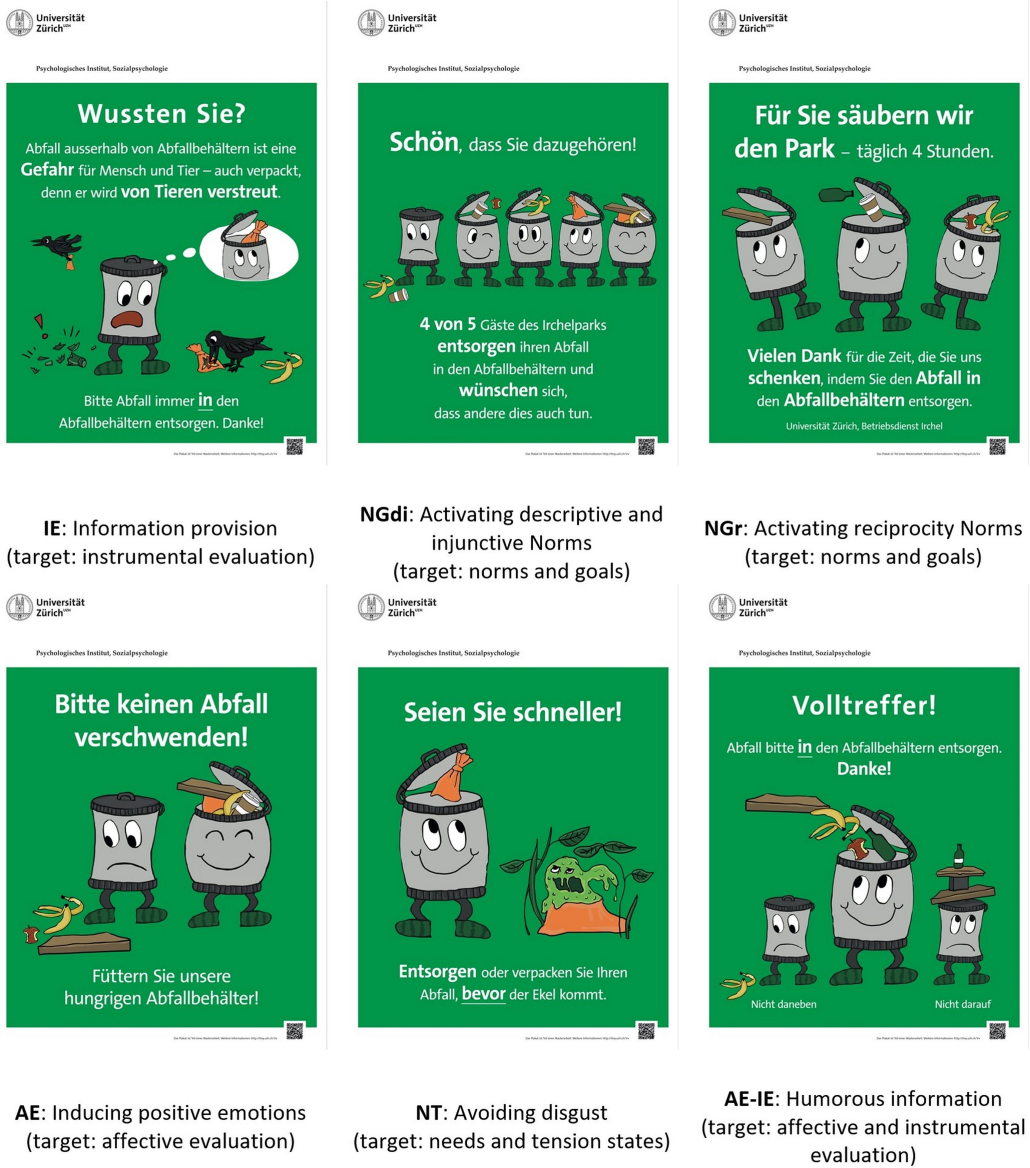
Experimental manipulations.

The experimental manipulations consisted of 1.2 × 0.8 m and 0.8 × 0.6 m large posters (Figure 3). If an intervention was active, according to the size and visibility within the zones, one to three equal posters were set up. From any position within a zone, at least one poster was visible. Each poster type targeted a specific dimension of behavioral determinants or, in one case, a combination of dimensions. The goal was to maximize the effect on one dimension of determinants and, at the same time, minimize the effect on all other dimensions.

The posters were created by a professional designer in close cooperation with the research team to guarantee the correct psychological effects. The theme of the campaign—sketched by the second author—are anthropomorphized garbage bins. The facial expressions were designed to convey much information, even without the viewer understanding the text but the figure did not have arms, so that the observer remains the only actor who can place waste in the bins.

While much of the message of the posters could be conveyed graphically, some posters required a larger amount of text to better specify and clarify the message. The text was provided in German, which is the official language of Zurich and the dominant language of the park visitors. Key words were highlighted allowing the message to be captured in less than a second. The posters comprised a QR-code that linked to the project page. There, general information about the project was provided, but no specific information about the posters.

Figure 3 presents all types of posters used in this study. The poster targeting instrumental evaluation (IE) conveys information about why littering is problematic. It states in German: “Did you know? Garbage outside the garbage bin is a threat to humans and animals – even if it is packed because animals can scatter it. Please always dispose of garbage in the garbage bins. Thank you!”

Two posters were used targeting norms and goals. NGdi activates descriptive and injunctive norms (“Glad you are part of it! 4 out of 5 guests of Irchelpark dispose of their waste in the waste containers and would like others to do the same.”), NGr targets the reciprocity norm (“For you we clean the park – 4 h a day. Thank you for the time you gave us by disposing of trash in the garbage bins. University of Zurich, Operational Service”). The information provided on these posters is based on a survey with park visitors in August to October 2018, and on information provided by the Operational Service.

The poster targeting the affective evaluation (AE) states: “Please do not waste any garbage! Feed our hungry garbage bins!” The idea was to put the observer in a positive mood with a funny message and, by doing so, help overcome the reluctance to carry the garbage to the garbage bin. This intervention is like the one used by Hansmann and Scholz (2003). To distinguish the effect of this poster from others, no meaningful information or reason is provided for the target behavior.

Regarding needs and tension states (NT), we focused on disgust. The idea was that the observers should dispose or pack material that, later, might become disgusting to touch. The message was: “Be faster! Dispose or pack your waste *before* the disgust comes.” While this poster, in the first place, provides information, it is expected to reduce experienced disgust and, therefore, affects the dimension of needs and tension states.

These five posters comprise the manipulations to provide the information for planning the large-scale campaign. In parallel to this preparative study, in Zone 7, an actual intervention with the goal to maximize behavior change was implemented. Therefore, the intervention in this zone does not follow the approach used for the rest of the preparative study. We include it in this paper, because it provides examples for a structural intervention, an intervention that targeted feasibility, and a poster design that targeted multiple dimensions to maximize the effect.

**Figure 4:**
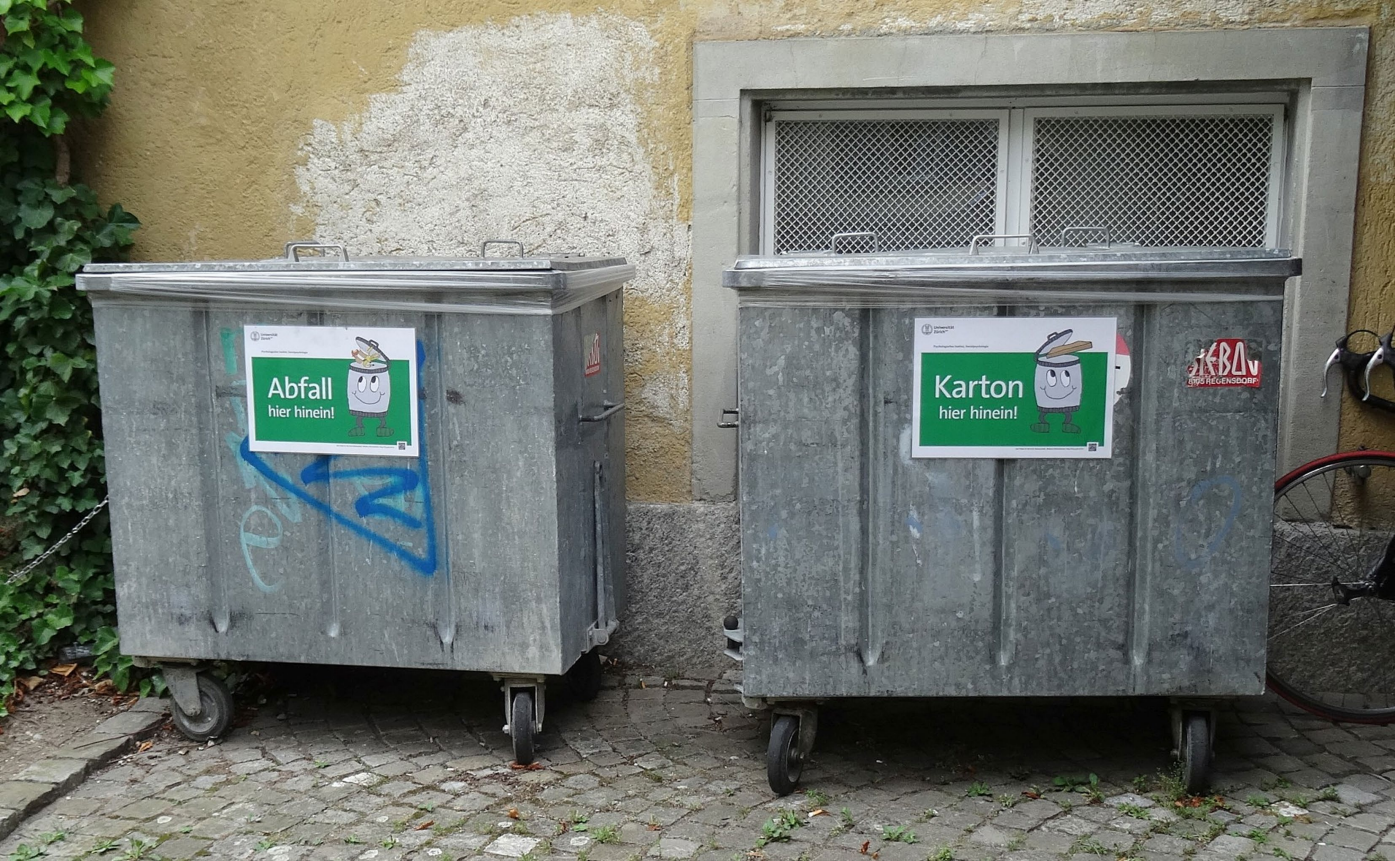
Structural measure: Second container to separate cardboard with signs ‘garbage in here’ and ‘cardboard in here’ (photo by first author).

The problem targeted in Zone 7 was that cardboard, particularly pizza boxes, were often put beside or above the garbage container. We assumed a possible reason for this is that people would like to separate cardboard for recycling, as they do at home. So, as a structural measure targeting feasibility, affordances in the form of a second container only for cardboard was provided and/or the Poster AE-IE was used. The poster stated: “Bull’s eye! Please dispose of waste *in* the waste garbage container. Thank you. Not next to it. Not on top.” This poster targets affective evaluation with the humorous eyecatcher, but by more specifically explaining the behavior, it also targets instrumental evaluation. We targeted these two dimensions, because we deemed it necessary to explain the behavior (IE), and previous research indicated that humorous prompts are more effective (Hansmann and Steimer, 2016). When two containers were placed, they were further clearly marked (Figure 4).

No intervention on consideration was implemented within this study. Due to the large number of garbage bins and the posters, when in place, forgetting as cause of littering can be ruled out. However, forgetting can be a critical factor for other behavior-change campaigns when such natural memory aids do not exist (e.g., Tobias, 2009).

#### 3.1.3 Experimental plan

The design of the preparative study is a between-subject longitudinal study with a rolling sample. The park was divided into seven investigation zones, as marked in Figure 2. These zones were isolated by ridges and vegetations, so that it was not possible to see the posters from other zones, when standing in one zone. These topographic features of the park limited the number of zones that could be specified for this investigation. It cannot be ruled out that people heading to a picnic spot passed through other zones and, thus, might have been influenced by the posters of these zones. However, the zones were specified in a way that no other intervention zone had to be crossed if individuals used the shortest route from the nearest park entrance to the respective zone. If the different interventions would have been confounded, it would have reduced the observed effects for all posters.

The design of the study (Table 1) comprises three intervention periods of 3 weeks, each. In-between the intervention periods, 2 weeks, and, before and after, 3 weeks without interventions were planned. However, due to bad weather, the baseline period was prolonged to 6 weeks. Due to the design of the analyses, this has no influence on the results, because all zones were affected equally from these conditions.

**Table 1:** Experimental plan.

	Week	Zone 1	Zone 2	Zone 3	Zone 4	Zone 5	Zone 6	Zone 7
May	19							
	20							
	21							
	22							
June	23							
	24	Control	*IE*	*NT*	Control	Control	*AE*	*Structural measure*
	25							
	26							
July	27							
	28							
	29	Control	*NGdi*	*NGr*	Control	Control	*IE*	*Structural measure and AE-IE*
	30							
	31							
August	32							
	33							
	34	Control	*NT*	*AE*	*NGdi*	Control	*NGr*	*AE-IE*
	35							
September	36							
	37							
	38							
	39							

The intervention abbreviations (e.g., IE) refer to the manipulations presented in Figure 3.

During the intervention periods, some zones remained without interventions to serve as control zones. Therefore, besides the special case of Zone 7, only in three (in the last period four) zones, interventions were applied—despite having five different interventions to apply. Consequently, not all interventions could be applied in one period. To control for effects of the period, in which an intervention was applied (e.g., due to varying weather conditions), and for differences in the zones (e.g., due to varying numbers and types of visitors), effects were always compared to the control zones of the same part of the park as the respective intervention zones.

All interventions were applied twice, but never in the same zone or phase (except for the interventions in Zone 7) to avoid confounding effects with characteristics of the zone or phase. While this design controls for biasing influences as much as is possible in the field, still certain confounding conditions cannot be excluded. Particularly, order effects are possible. For example, a very effective intervention might reduce littering so much that a following intervention cannot reduce littering anymore and, thus, appears to have no effect—or the effect of a following intervention might only result due to the intervention that was applied before. This will be considered in the interpretation of the results.

#### 3.1.4 Data gathering

All analyses presented here are based on ‘objective’ measurements of litter, avoiding problems with subjective assessments of the amount of litter or dirtiness of a place. Every day—except in particularly bad weather when almost no one was present in the park (28 out of 147 days)—litter was measured, starting late afternoon. Because picnic littering happens mainly from noon to the evening and the park is cleaned in the morning, litter measured in this period should include most of the picnic littering but exclude most party littering, which occurs mostly at night.

Litter measurement followed a strict protocol: In each of the zones (see Figure 2), 3 to 16 measurement points were specified, and, whenever litter was measured, all 55 measurement points were considered. The measurement points were kept unchanged for the entire study and were defined based on locations where littering behavior was expected. We selected every garbage bin in the zone (to measure garbage deposited outside the bin), and locations where people tend to stay longer time (e.g., benches). At each measurement point, the students who did the measurement specified one square meter—the one with most litter or the one they checked previously—and collected all garbage within this square meter. So, which square meter was searched for garbage changed over time but was always within the area of the pre-defined measurement point. For example, a measurement point could be a bench. On 1 day, the students might have collected garbage from a square meter on the table, on another day, the square meter might have been beside the bench on the ground. The reported quantities of litter were always volume, without considering the weight or type of garbage. Volume as indicator was selected because it is closest to the visual impact litter has. Weight would strongly bias the measurement towards overestimating the impact of litter made from glass or metal. Volume might lead to overestimating larger over smaller items, which was desired, because large items are more visible, also from greater distance. The garbage was filled in bags to determine the volume—or directly measured, if larger volumes were encountered (e.g., shopping bags filled with garbage). Because volume depends strongly on the compression, the instruction was to keep the form of the garbage collected as much as possible (i.e., to compress as little as possible). Nevertheless, larger and harder pieces of garbage (e.g., bottles) might have been somewhat overestimated compared to smaller and softer variants (e.g., paper). After reporting the volume, the garbage was correctly disposed.

The number of data points was completely determined by the structure of the zones—limiting the maximum amount of possible measurement points—and the time available to investigate littering—limiting the number of days to measure litter. Data were gathered during the entire study period and only halted in case of particularly bad weather. Therefore, no a-priory power analyses were performed.

#### 3.1.5 Data analyses

Within the preparative study, it was planned to investigate some basic-research questions. The pre-registered (https://doi.org/10.17605/OSF.IO/52C3A) hypotheses relate effects of the posters to changes in psychological constructs assessed with questionnaires. However, because in too many zones too few data could be collected by questionnaires, these analyses could not be performed. The presented research is purely explorative and omits questionnaire data.

In general, littering was low (82.9% of values were zero), however, on some days, at some measurement points, large amounts of litter were encountered (0.7% of the measurements have values >10 L, with up to 277 L). This distribution did not allow any traditional statistical approach and, thus, the data were analyzed differently. The effect of interest is the difference between the change of litter due to an intervention compared to the change of litter in the control condition (i.e., how much more litter was reduced in the intervention zone than in the control zone). A straightforward approach to test this with the encountered irregular distribution is to bootstrap this difference of changes. We bootstrapped the 90% confidence intervals to estimate the range of effects with *p* < 0.05 for a one-sided hypothesis of reducing the amount of litter. The analyses were done with R Project for Statistical Computing (RRID:SCR_001905). The data and scripts for these analyses are provided on https://osf.io/s32qp/.

We calculated the average of the measures during the intervention (short-term effect) or after the intervention (long-term effect) in the target zone and the structurally most similar control zones. The between-zone difference of the within-zone changes provides the value of the effect. For example, to test the effect of the intervention in Zone 3 in Phase 4, we bootstrapped the differences of the change of means from Phase 3 to Phase 4 (short-term) and Phase 3 to Phase 5 (long-term) in Zone 3 (intervention) and Zone 1 (control):


ShortTermEffect43=meanVolumeZone3Phase4−meanVolumeZone3Phase3−meanVolumeZone1Phase4−meanVolumeZone1Phase3

LongTermEffect43=meanVolumeZone3Phase5−meanVolumeZone3Phase3−meanVolumeZone1Phase5−meanVolumeZone1Phase3

With this design, we controlled for confounding effects, for example, due to phases with generally more or less produced garbage in the park. By using the mean (instead of the sum) of measures per zone, we also control for possible effects due to the number of measurement points. Still, order effects cannot be ruled out. These cannot be controlled for, within the analysis, but were controlled qualitatively based on the resulting effects. An increase of the effects over time would indicate that previous interventions might be an important factor for later effects. A decrease of effects could indicate that the amount of littering was so much reduced by previous interventions that too little litter was present for later interventions to be able to show effects.

For testing whether enough litter was present before the interventions to identify an effect, we calculated the potential for change, which expresses how much a perfect intervention could reduce litter. This potential is calculated by adding the change in the control zone to the amount of litter in the intervention zone before the intervention. Thus, the potential for change expresses the amount of litter in the intervention zone assuming a change equal to that of the control zone. For the above-mentioned example of long-term effect, the intervention potential would be:


LongTermInterventionPotential43=meanVolumeZone3Phase3+meanVolumeZone1Phase4−meanVolumeZone1Phase3

### 3.2 Results

Litter was measured on 119 out of 147 days, leading to 6,536 measurements. Of these, 82.9% had a value of 0.0 L, 14.3% had a value between 0 and 1 L, 1.1% had a value between 1 and 2.5 L, 1.0% had a value between 2.5 and 10 L, and 0.7% had a value of more than 10 L up to 277 L. So, in general, littering was low, and the total amount dominated by some rare high values. Table 2 compiles the average amounts of litter measured per measurement point in the different zones and phases, and Table 3 compiles the bootstrapped differences of changes.

**Table 2:** Average liters of litter collected per measurement point in the different zones and phases.

	*n* of MP	Phase 1	Phase 2	Phase 3	Phase 4	Phase 5	Phase 6	Phase 7	Total
*n* of MP		21	18	12	18	13	20	17	119
Zone 1	9	0.056	0.235	0.546	0.196	0.392	0.149	0.238	0.232
Zone 2	10	1.176	0.748	0.197	1.250	0.203	0.298	1.928	0.877
Zone 3	8	1.800	1.209	5.582	0.630	0.138	0.107	0.094	1.210
Zone 4	4	1.571	1.444	0.013	0.433	0.088	0.057	0.021	0.585
Zone 5	5	0.010	0.048	0.052	0.111	0.182	0.137	0.146	0.095
Zone 6	16	0.144	0.066	0.009	0.102	0.020	0.061	0.094	0.077
Zone 7	3	0.113	0.075	0.014	0.007	0.067	0.108	0.181	0.085
Total	55	0.649	0.483	0.946	0.421	0.153	0.134	0.455	0.451

*n of MP indicates the number of measurement points per zone and phase, respectively.*

**Table 3:** Bootstrapped differences of changes of measured litter (averages per measurement point) in liters.

		Zone		Short-term				Long-term			
Intervention	Phase	I	C	Pot.	Est.	LL	UL	Pot.	Est.	LL	UL
IE	2	2	1	1.36	−0.61	−1.95	1.02	1.67	**−1.47**	**−2.58**	**−0.06**
IE	4	6	5	0.07	0.03	−0.17	0.19	0.14	**−0.12**	**−0.22**	**−0.02**
NGdi	4	2	1	−0.15	*1.40*	*0.55*	*2.15*	0.04	0.16	−0.36	0.76
NGdi	6	4	5	0.04	0.01	−0.16	0.19	0.05	−0.03	−0.21	0.17
NGr	4	3	1	5.23	−4.60	−8.79	0.47	5.43	**−5.29**	**−9.59**	**−0.61**
NGr	6	6	5	−0.02	0.09	−0.06	0.23	−0.02	0.11	−0.05	0.27
AE	6	3	1	−0.11	0.21	−0.21	0.52	−0.02	0.11	−0.42	0.46
AE	2	6	5	0.18	−0.12	−0.26	0.05	0.19	**−0.18**	**−0.30**	**−0.02**
NT	6	2	1	−0.04	0.34	−0.09	0.71	0.05	1.88	−1.54	3.79
NT	2	3	1	1.98	−0.77	−3.06	1.46	2.29	3.29	−1.87	7.87
Structural	2	7	5	0.15	−0.08	−0.20	0.05	0.16	**−0.14**	**−0.23**	**−0.04**
Str. and AE-IE	4	7	5	0.07	−0.07	−0.16	0.05	0.14	−0.08	−0.18	0.02
AE-IE	6	7	5	0.02	0.09	−0.26	0.23	0.03	0.15	−0.03	0.31

Short-term, intervention phase–pre-intervention phase; long-term, post-intervention phase–pre-intervention phase; I, intervention zone; C, control zone; Pot., potential for change; Est., estimate; LL, lower limit; UL, upper limit of the 90% confidence intervals (bootstrapped with 1,000 replicates); The intervention abbreviations (e.g., IE) refer to the manipulations presented in Figure 3. Bold font highlights statistically significant effects with *p* < 0.05 one-sided, expecting a reduction. Italic font highlights statistically significant effects with *p* < 0.05 two-sided, in the case of an (unexpected) increase in litter.

Table 3 shows that, short-term (i.e., while the interventions were set up), none of the interventions reduced litter significantly. The only statistically significant effect is an *increase* in measured litter for the first NGdi intervention (estimated effect = 1.40, 95% CI = [0.37, 2.29]). However, long-term (i.e., comparing values of the control phase directly after the interventions to before the interventions), both information-based posters in Zones 2 and 6 (IE, estimated effects −1.47 and −0.12), the activation of the reciprocity norm in Zone 3 (NGr, estimated effect = −5.29), the affect-oriented poster in Zone 6 (AE, estimated effect = −0.18), and the structural measure in Zone 7 (estimated effect = −0.14) all reduced litter significantly.

Table 3 also shows that the potential for change, in some cases, was small or even negative (6 of 13 changes had a potential <0.05 L). For most non-significant long-term effects, the potential for change was 0.05 L or smaller. This lack of effects in some cases might be due to a lack of litter that the interventions could have reduced. Of the remaining two non-significant long-term effects with a potential for change >0.05 L, the combined structural and AE-IE intervention had a marginal non-significant effect in the expected direction (estimated effect = −0.08). Only the intervention on disgust (NT) was clearly non-significant and even shows a tendency towards an increase in litter (estimated effect = 3.29).

The interventions with significant long-term effects reduced littering by 86–97% of the potential for change, while the marginally non-significant combined structural and AE-IE intervention reduced littering by 54%. Thus, except for the intervention on disgust and on injunctive and descriptive norms, all measures lead to considerable long-term effects. Due to the small potentials for change for both zones in which the intervention on injunctive and descriptive norms were applied, no conclusive results for the effect of this intervention on reducing littering can be derived.

Regarding order effects, Table 3 shows that in Zone 2 the first intervention had a significant effect and for all further intervention the potential for change was <0.05. In Zone 3, the first intervention had no significant effect, while the second intervention had a significant effect. Then, again, for the last intervention, the potential for change was <0.05 L. In Zone 6, the first intervention had a significant effect, but also the second intervention in Phase 4. For the third intervention, the potential for change was, again, < 0.05 L. Finally, in Zone 7, the first intervention had a significant effect, and the second a marginally non-significant effect. In Phase 6, the potential for change was <0.05 L. We conclude that an order effect exists with the first interventions having more chances to reach high effect strengths. Nevertheless, not all interventions in the first wave of interventions showed significant effects and some interventions in the second wave of interventions had significant effects. An increase of effects over time due to adding up of intervention effects was not observed, but the potential for change resulted in a powerful indicator for the interpretability of results. Note that, for all interventions except NG_di_, the effects could be estimated due to a large-enough potential for change, at least once. For NG_di_, a significant negative short-term effect (i.e., increase of litter) was found, indicating potential problems with this intervention.

## 4 Large-scale campaign

### 4.1 Introduction

The preparative investigation identified processes that could be targeted by campaigns to reduce littering. Most psychological studies stop at this point and conclude that the results can be used to design future interventions. This might give the false impression that it is a small step from having quantified effects to launching a behavior-change campaign. However, the development of a large-scale campaign is an enormous endeavor that goes far beyond upscaling preparative investigations. While not generalizable, we provide a brief description of how the large-scale campaign was designed based on the preparative study.

First, the problem a campaign shall mitigate needs to be revised based on the data gathered during the preparative study—which is much more than the data used in the quantitative analyses. Any piece of information, even anecdotal observations, can be helpful to improve the campaign design. In the present case, it was observed that the collected litter around the garbage bins was often torn into small pieces. This indicates that animals in the park rather than humans distributed this garbage. Also, it was observed that garbage was frequently placed in bags beside the garbage bins, sometimes because the garbage bins were full and sometimes, whilst the bins were empty. Together with the overall low level of littering in the park, the goal of the large-scale campaign was not so much to reduce littering, in general, but to reduce depositing garbage besides garbage bins.

While the preparative study focused on psychological measures, the campaign design also comprised structural components, particularly setting up additional garbage containers at locations that the preparative study identified as critical. Providing options to collect garbage fractions separately was proposed but rejected due to the high costs involved. Finally, a psychological measure was developed that prompted people not to deposit garbage beside the bins but to take it to the next container, if it did not fit in the bin.

Because the posters of the preparative study were designed to target one specific psychological dimension each and littering in general, the poster for the large-scale campaign was designed from scratch. Based on the result of the preparative study that particularly targeting instrumental and affective evaluations reduced littering, while targeting norms and tension states had rather unclear effects, the poster was designed to inform in a funny way and to avoid any appeals to injunctive norms. The poster was designed to visualize the problem of animals distributing garbage deposited beside the bins, as well as the solution of depositing garbage in large containers. Text was only used to clarify the message and to provide humorous notes. Finally, additional information that supports correct garbage disposal was provided. In 2022, the campaign was ready to be launched.

### 4.2 Materials and methods

**Figure 5:**
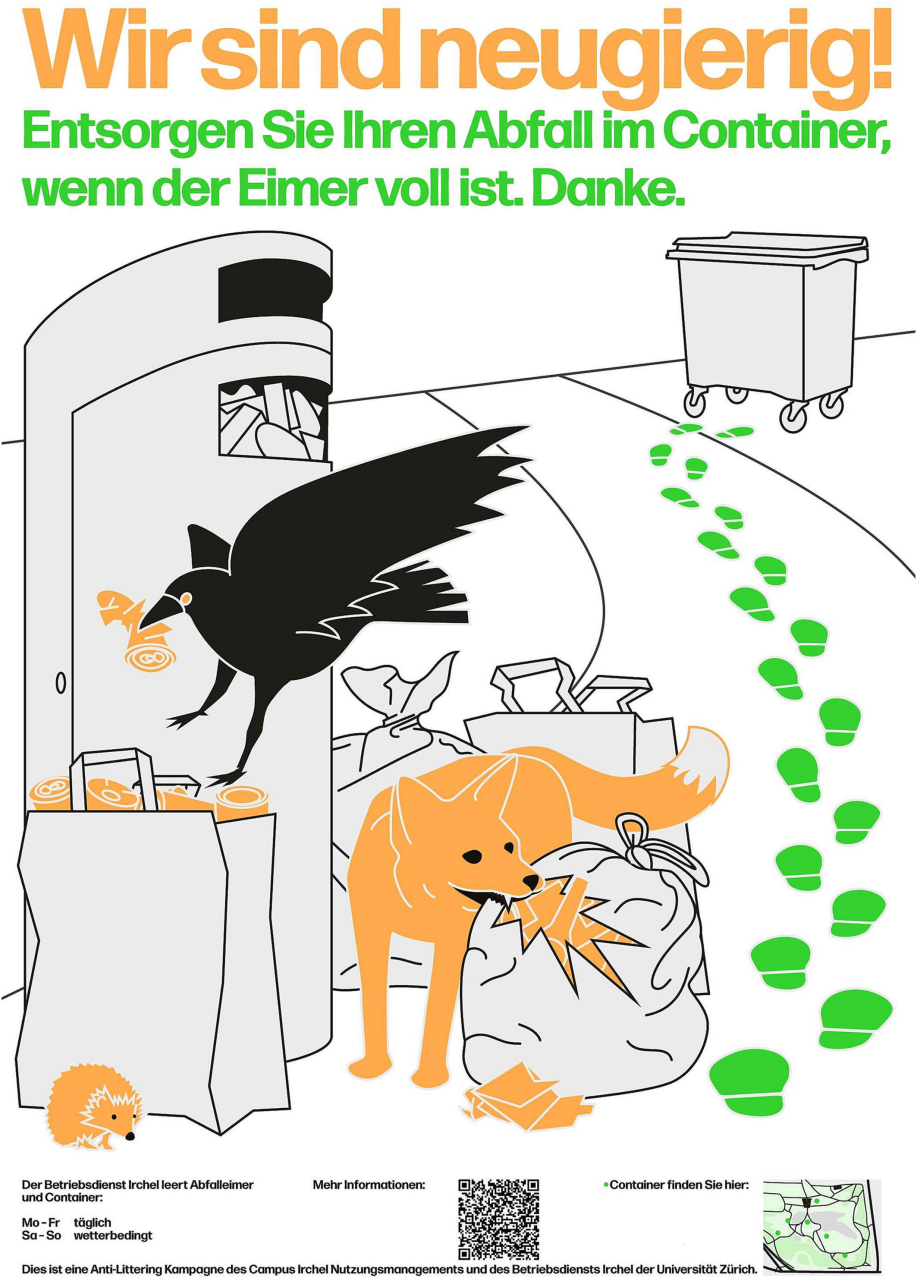
Poster of the follow-up campaign.

The poster used for the campaign is presented in Figure 5 and follows the design principle explained in the introduction. It reads: “We are curious! Dispose of your waste in the container when the bin is full. Thank you.” The message should have been clear already by the picture, but complementary information was provided by the title text and at the bottom, such as, the locations of the large waste containers and the hours when the garbage is collected. A QR-code linked to the webpage of the campaign with further information. The poster was designed with only two colors that represented the problem (orange) and solution (green). The rest of the poster was mostly white with some gray to contrast with the colorful background of the park.

The campaign’s budget only comprised funds for the intervention material. Nevertheless, we wanted an estimate of the campaign’s impact. Therefore, data gathering and analysis were designed to maximize the information obtainable and minimize the effort. Data gathering consisted of photos taken from selected garbage bins by the Operational Service team. This was done with the maintenance personnels’ smartphones, but still, taking off the work gloves, preparing the telephone, taking the pictures, and putting the work gloves back on consumed valuable time. Therefore, the number of garbage bins monitored and the period of data gathering had to be limited. We agreed on monitoring 11 garbage bins for 13 weeks. Two photos per bin were taken from orthogonal directions before they were emptied, and the area was cleaned up, what usually happened twice a day (in the early morning and shortly after noon). The photos were analyzed by a student assistant, who estimated the volume of garbage beside the garbage bins based on systematic estimation guidelines and co-checked by one of the authors.

To get something like a control condition, around 5 garbage bins, no posters were placed. Besides these 5 ‘control’ bins, 6 bins that had posters beside them were monitored. As baseline and post-intervention phases, the monitoring started 4 weeks before the campaign started and ended 3 weeks after the campaign ended. Therefore, in weeks 5 to 10 of the monitoring period, posters were installed. This data-gathering design allowed the data to be analyzed as in the 2019 preparative study presented above. To exclude periods with particularly bad weather and some days with unusual events (e.g., the SOLA relay race), for the pre-intervention period, only Days 15 to 26 (72 data points in the intervention and 60 in the control condition) were analyzed, and for the intervention period, Days 43 to 71 (174 data points in the intervention and 145 in the control condition) were analyzed. For the post-intervention period, Days 76 to 92 (102 data points in the intervention and 85 in the control condition) were considered in the analysis.

As mentioned, instead of area-wide picnic littering, the campaign focused on reducing garbage explicitly deposited beside the garbage bins. Because both kinds of littering can happen around the garbage bins, we used the amount to distinguish them, assuming that large volumes (e.g., bags with litter, pizza cartons) are the kind of litter we are interested in reducing. Therefore, the analyses were conducted with all the raw data first, and then with values between 0 and 40 L recoded to 0, while larger values remained raw measures. The cut-off value was set to 40 L, because data explorations showed the strongest effect for this value, although only 23 measures (3.6%) were larger than zero. So, when interpretating the results, it must be considered that the estimated effects depend on this context-specific cut-off value.

### 4.3 Results

With all the raw data considered in the analysis, like in the preparative study, no short-term effect was found (average difference of changes = 0.012 L, 90% CI = [−3.412, 3.458]). The long-term effect shows a non-significant tendency towards a reduction in littering (average difference of changes = −0.416 L, 90% CI = [−2.55, 2.145]). Again, most measures (88.2%) had values of 0, but sometimes also large values up to 702 L were reported. More than 25 L was measured in 3.3% of the cases.

When raw values up to 40 L were set to 0 (to exclude area-wide littering) a relevant long-term effect of −1.54 L (90% CI = [−3.09, 0.74], 80% CI = [−3.09, −0.04]) was identified. Identifying such a positive tendency is a promising result, considering the few data available and the challenges of investigations in applied settings.

## 5 Discussion

This paper presented a theoretical tool and methodological approach for designing and evaluating large-scale campaigns based on scientific evidence and methods. This approach was used to develop a large-scale campaign in an urban park, and to estimate its impact on littering. We will start with discussing the results of the preparative study and the campaign evaluation, then discuss the methodological approach and theoretical tool, and finish the discussion with limitations and the practical implications of this paper.

### 5.1 Discussion of the results of the preparative study and the campaign evaluation

The preparative study was implemented as a quasi-experimental field study with experimental manipulations designed to have effects on one dimension of behavior determinants and not on others. The volume of litter was measured daily in three to four intervention and two to three control zones. By bootstrapping the differences of *changes* in the volume of litter in the intervention compared to the respective control zones, the effects of the interventions were estimated. This way, the importance of the different dimensions of behavior determinants in the specific location and the specific target population could be assessed without using surveys.

In the preparative study, no intervention showed significant effects in the expected direction when in place (i.e., short-term effects). However, several interventions reduced litter in the phase without intervention directly after the respective intervention phase compared to before this intervention. Such patterns were also found in other studies (e.g., Sibley and Liu, 2003). The reason for this might be that even behind the narrow specification of littering as picnic littering, many behaviors, such as preceding actions, like bringing along bags to collect garbage or not bringing single-use materials, are involved, that, finally, lead to solid waste being misplaced. Since people might only visit the park infrequently, even if the intervention was effective, it might take days or weeks to observe a change in the amount of litter, which might be observed only after interventions were already removed.

Significant long-term effects were found for the interventions targeting instrumental evaluation (information provision), norms and goals (activating reciprocity norms), affective evaluation (inducing positive emotions), and the structural feasibility measure (additional garbage container to dispose of cardboard separately). In most cases where no significant effect was observed, it could be attributed to not enough litter being in the respective zones to be reduced by the interventions. Here, an order effect was identified: In two out of three zones, after an intervention with significant effects, the potential for change was too small for the next intervention to be able to achieve any effects. Nevertheless, all interventions except the poster targeting descriptive and injunctive norms, could be tested with a large-enough potential for change – and not all resulted in significant effects. Particularly, the intervention on disgust did not reduce litter, despite having a large potential. Neither did the accompanying poster increase the structural measures’ effect.

During the intervention phase, the only statistically significant effect was an *increase* of litter in a zone where injunctive and descriptive norms were targeted. This does not mean that these norms are not important determinants of litter avoidance. Particularly in questionnaire-based studies, norms mostly have a strong explicative power (e.g., Oduro-Appiah et al., 2024; Ojedokun et al., 2022; Farage et al., 2021). It even might be that the appeal to injunctive and descriptive norms in our study has been too strong, and thus lead to reactance (Brehm, 1966) like those found for explicit commands in other studies (e.g., Reich and Robertson, 1979). Also, Gangl et al. (2022) found an increase of littering when using appeals to injunctive norms. However, an increase of litter was not found in the second zone where the same posters were set up, neither was it found when comparing the post- and pre-intervention phases, which might indicate that the effect was incidental. So, the effect of appealing to injunctive and descriptive norms remains inconclusive in our study.

A confounding effect due to applying different interventions simultaneously is improbable, due to the strong effects found for successful interventions, which showed between 86 to 97% of their theoretical maximal effect. If the intervention effects had diffused into other zones, particularly the control zones, the effects would have been weaker or absent. Order effects regarding the reduction of the potential of change were already discussed. However, could later effects be partially caused by previous interventions? In Phase 4, two posters had significant effects. In Zone 3, NGr had a significant effect after the ineffective NT intervention. While improbable, it cannot be ruled out that the effect of the NGr intervention is only achieved by having previously a NT intervention. In Zone 6, IE was applied after AE, both with significant effects. However, IE also had a significant effect in Phase 2 (in Zone 2) and, thus, the effectiveness of this poster appears to be independent of previous interventions.

Besides creating the basis for designing the large-scale campaign, a small campaign was run during the preparative study in Zone 7. The structural measure on feasibility by providing a second garbage container for collecting cardboard only, reduced littering in this zone significantly. An additional psychological intervention—a poster targeting the instrumental and affective evaluations by providing information in a funny way—appears to rather have reduced the effect of the structural measure. This leads to the conclusion that some littering is done intentionally to separate certain fractions of waste for recycling by the cleaning personnel. Therefore, providing the possibility to separately collect such waste fractions can reduce littering, indicating also an important role for feasibility and structural measures.

Based on the results of the preparative study, a large-scale campaign was developed and implemented. The posters that targeted a reduction of garbage deposited beside the garbage bins were designed mainly to provide information and induced positive emotions. While less data could be gathered than in the preparative study, the same analyses could be performed as for the latter. A reduction of larger amounts of garbage close to garbage bins was identified with *p* < 0.10 (one-sided, expecting a reduction). Considering the few data that could be gathered in this applied campaign, this result is promising and supports the theoretical and methodological approach presented.

### 5.2 Discussion of the approach in the light of differences between basic research and applied settings

The theoretical approach challenges the view of psychological constructs being specified by their operationalization as questionnaire items. It is not questioned that surveys are, in many situations, the most efficient form for gathering data from the target population and that these data can be valuable, also for designing campaigns. However, for the problem of designing behavior-change interventions, specifying constructs based on their expected role in changing behaviors is preferable. The proposed six dimensions are an assumption based on evidence found in literature and campaigning experiences but cannot be tested like a model or hypotheses. Rather, the proposed concept must prove that it is functional for designing successful campaigns.

It is open to discussion whether more, less, or different dimensions might lead to better campaign designs, but some rules the proposed dimensions are based on are general: Interventions targeting different dimensions should follow different design principles and, sufficiently often, not be applied together because of conflicting effects of the different dimensions. In turn, interventions targeting the same dimension should follow similar design principles and vary mainly in the content (e.g., whether an argument is about costs in money or in time). Further, the number of dimensions should not be too large and, maybe most importantly, for all dimensions it should be clear what is not included but part of other dimensions. The preparative study allows discussing these general points more specifically. A good example is the norms-and-goals dimension. Interventions on three different norms were applied, with two norms covered on a single poster. Why are these all placed on a single dimension? All three interventions refer to a socially desired behavior, without conveying any reasons of why the respective behavior would be desirable. While the effectiveness of different references to norms can vary, we still assume that similar psychological processes are triggered. It might make a difference, whether norms are conveyed by reference to others’ behavior or others’ opinions. However, knowing about many people performing a behavior might strongly affect what a person thinks about the opinions of others, and the other way around. Therefore, all these interventions are considered targeting the same dimension.

For discussing the separation of dimensions, the difference between affective evaluation and needs and tension states is a good candidate. Both dimensions refer to affective states or emotions and, thus, combining them, as is done in most behavior models, might be obvious. However, interventions targeting these dimensions are very different. While affective-evaluation interventions mainly associate affective states with certain behaviors, interventions targeting cognitive tension states mostly provide information and arguments. These information and arguments target emotions, in contrast to information and arguments targeting costs and benefits for interventions on the instrumental evaluation dimension, or, for example, normative aspects for the norms-and-goals dimension. The striking difference in how these two dimensions are addressed by interventions requires distinguishing affective evaluation and needs and tension states. It is also important to note that, while needs and tension states affect behavior even with close to no cognitive effort involved, the creation and change of them might require considerable cognitive resources.

For data gathering, we proposed a quasi-experimental approach with experimental manipulations that target a single dimension of behavior determinants and affect as little as possible other dimensions. By measuring changes in the consequences of the behavior, such as the amount of litter, the relevance of the different dimensions for changing the behavior can be estimated. This approach omits surveys and behavior observations, but still allows conclusions about psychological processes involved. Nevertheless, it comes with its own challenges. The number of experimental conditions is limited by the specific setting and, due to the limited controllability, certain confounding or biasing effects cannot be ruled out. In the preparative investigation, we found no evidence for confounding effects due to applying several interventions in parallel, but order effects were identified, which made the estimation of effects for some later implemented interventions impossible. Such field investigation might not come close to laboratory experiments, but the advantage of measuring effects *in-situ* with the actual target population compensates for this.

A larger issue with the proposed approach might be seen in the question of validity, and how it can be known whether the posters actually addressed the theoretical dimensions. This is, however, a problem with any measurement. It remains also unknown whether a scale in a questionnaire assesses the construct it is meant to assess. Arguments to defend such instruments are based on expert opinions and conventions. Therefore, the same justification is used for the experimental manipulations used in the preparative study. If there is no reason to assume that these posters had effects on other dimensions, the experimental manipulations are judged valid. In fact, the validity problem is far less problematic with the proposed approach than with questionnaire-based measurements, because the process of generating the conclusions for the campaign are the same as the processes intended to trigger by the campaign. If a poster with a specific design principle shows an effect in the preparative study and in the campaign the same design principle is used, the campaign could be successful even if the manipulation influenced a different dimension than assumed. In the case of questionnaire-based measures, such error could be fatal. For example, if attitudes are identified as critical, but the measure is mainly influenced by normative considerations, a campaign targeting attitudes would fail. Of course, both data-gathering approaches could be combined, and the data correlated—what we planned to do in the preparative study, but the surveys failed to deliver useful data. However, if the correlation is low, it would remain unclear, whether the problem lies in the experimental manipulation, the questionnaire measure, or different issues.

Finally, it is important to highlight the differences between the preparative study, which is considerably closer to common field research, to the large-scale campaign. First, the final intervention is very different from the experimental manipulations used in the preparative study. As already mentioned, the latter was not designed to maximize behavior change or find the intervention to maximize such impact. The study targeted an understanding of psychological processes relevant for a behavior-change campaign. The large-scale campaign was then designed based on all the information compiled during the preparative study—not only the effect estimates, but also, for example, rather anecdotal evidence on the litter collected. Another important difference is the limited possibilities for gathering and analyzing data within large-scale campaigns. Surveys are often difficult and even simple measurements, such as taking photos, must be limited to a minimum. Nevertheless, the proposed approach for data gathering and analysis allowed a rough estimation of the campaign effect.

### 5.3 Strengths and limitations

While this study contributes to the evidence on possible determinants of litter avoidance behavior and effects of large-scale litter-reduction campaigns, its contributions lay mostly in the applied sector. The proposed theoretical and methodological approach proved to be feasible for designing and evaluating large-scale campaigns. We demonstrated how behavior should be specified from a psychological perspective and we translated questionnaire-oriented construct specifications into intervention-oriented dimensions. This theoretical concept allows investigating behavior determinants without depending on surveys and supports the development of behavior-change campaigns. However, even when working with this tool, the step from a preparative investigation to a large-scale campaign is huge. We described some considerations for such a step, but these might vary from campaign to campaign. Therefore, the main goal of demonstrating the feasibility of the proposed approach for evidence-based design of campaigns was achieved.

Nevertheless, some limitations need to be discussed. Particularly challenging was the distribution of littering. For most days and measurement points, no or very little litter was measured, but some measurements revealed enormous numbers. While the lack of a constant rate of baseline littering makes the statistical detection of a reduction of littering due to interventions difficult, such data distributions might be frequent, in the case of littering. Also, other studies encountered frequent low amounts of littering (e.g., Bator et al., 2011; Gangl et al., 2022) and subjective estimates of littering might be overestimated due to litter being highly salient, while the absence of litter is hardly noticed. A pile of garbage 1 day might lead to the conclusion that an entire park has a littering problem, despite having no litter on most days and at most locations. Therefore, analysis methods are required that can deal with such data distributions—and too smooth data distributions might even be questioned.

Further limitations come from the already several times mentioned limited controllability of studies in field settings. It cannot be ruled out that the effect of the campaign posters might be moderated by the differences of the zones and the populations visiting them. In turn, it is possible that the manipulations had effects in zones other than those they were used in. Because clear differences between the zones could be identified and the effects are rather strong, the latter problem might not have been present in this study.

Finally, for the preparative study, further investigations were planned and pre-registered. These investigations were based on questionnaire data, which could not be gathered in an adequate form. Therefore, the pre-registered analyses could not be realized.

### 5.4 Practical implications

For practice, the most important contribution is the introduced approach to identify the critical determinants for changing a behavior in a specific setting. Whenever several comparable but isolated zones can be specified, the proposed approach can be used to gather the information, based on which behavior change campaigns can be designed for a specific location and target group. While the posters (or other types of manipulations) need to be adapted to the specific situation, conceptually, the study could be applied to any place and other behaviors to identify critical determinants. However, the proposed approach requires a different theoretical basis than common basic-research studies. A specification of a relatively small number of classes or dimensions of behavior determinants is required, which are clearly distinguishable regarding how they are manipulated. Testing whether a certain construct, such as attitudes, determine behaviors or behavior changes, is very different from exploring what determines the change of a behavior, for which a campaign is developed.

With respect to littering, the results indicate three promising determinants on which campaigns can focus to reduce littering. First, the effect of structural measures was reconfirmed. Further, providing information on the problem and inducing more positive emotions related to littering-reducing behaviors appear to help reduce littering. Norms appear to be important factors for littering reduction, but direct norm appeals might not have the desired results. In our study, activating injunctive and descriptive norms led to an increase of littering, maybe due to reactance. So, less explicit measures to activate norms, such as reducing litter to convey the descriptive norm or the use of models for injunctive norms, might be more fruitful (but also much more expensive). Also appealing to reciprocity norms led to desired effects. In the investigated setting, the posters that targeted the tension state of disgust did not have any effect. This might be due to visitors in this park already wrapping garbage before it becomes disgusting, thus, this measure might work in other settings. However, it is also possible that disgust is not a relevant factor for reducing littering.

More generally, we advocate for theoretical and methodological approaches that are better suited to deal with problems of applied settings. While basic research continues to be the foundation of applications, the requirements and challenges of applied settings are different from the ones of basic research. In turn, it would also be important to consider evidence collected in applied settings in theory building. While confounding effects cannot be fully ruled out and data quality is sometimes low, repeatedly successfully designed campaigns would be a strong indicator that psychological processes were well identified and play an important role in changing the targeted behavior.

## Data Availability

The datasets presented in this study can be found in online repositories. The names of the repository/repositories and accession number(s) can be found at: OSF https://osf.io/s32qp/.

## Footnotes

^1^Within this investigation, we only consider processes in the situation of the behavior selection (i.e., when a behavior is performed or not). If barriers to behavior change are personal, more enduring knowledge, beliefs, opinions, values, etc. need to be changed. Barriers related to the population hinder behavior change due to unfavorable dynamics in the target population, for example, a lock-in due to nobody wanting to be the first to change the behavior, even though most people would prefer a change in behavior. Further, barriers to behavior change can also be structural, mainly due to rules (e.g., laws), economic conditions (e.g., prices), or the provision of infrastructure and services (e.g., lack of garbage bins).
